# Roles of Heat Shock Proteins in Apoptosis, Oxidative Stress, Human Inflammatory Diseases, and Cancer

**DOI:** 10.3390/ph11010002

**Published:** 2017-12-23

**Authors:** Paul Chukwudi Ikwegbue, Priscilla Masamba, Babatunji Emmanuel Oyinloye, Abidemi Paul Kappo

**Affiliations:** 1 Biotechnology and Structural Biochemistry (BSB) Group, Department of Biochemistry and Microbiology, University of Zululand, KwaDlangezwa 3886, South Africa; pikwegbue@yahoo.com (P.C.I.); presh4u@rocketmail.com (P.M.); tunji4reele@yahoo.com (B.E.O.); 2 Department of Biochemistry, Afe Babalola University, PMB 5454, Ado-Ekiti 360001, Nigeria

**Keywords:** apoptosis, cancer, heat shock proteins, inflammation, reactive oxygen species, tumour necrosis factor-α

## Abstract

Heat shock proteins (HSPs) play cytoprotective activities under pathological conditions through the initiation of protein folding, repair, refolding of misfolded peptides, and possible degradation of irreparable proteins. Excessive apoptosis, resulting from increased reactive oxygen species (ROS) cellular levels and subsequent amplified inflammatory reactions, is well known in the pathogenesis and progression of several human inflammatory diseases (HIDs) and cancer. Under normal physiological conditions, ROS levels and inflammatory reactions are kept in check for the cellular benefits of fighting off infectious agents through antioxidant mechanisms; however, this balance can be disrupted under pathological conditions, thus leading to oxidative stress and massive cellular destruction. Therefore, it becomes apparent that the interplay between oxidant-apoptosis-inflammation is critical in the dysfunction of the antioxidant system and, most importantly, in the progression of HIDs. Hence, there is a need to maintain careful balance between the oxidant-antioxidant inflammatory status in the human body. HSPs are known to modulate the effects of inflammation cascades leading to the endogenous generation of ROS and intrinsic apoptosis through inhibition of pro-inflammatory factors, thereby playing crucial roles in the pathogenesis of HIDs and cancer. We propose that careful induction of HSPs in HIDs and cancer, especially prior to inflammation, will provide good therapeutics in the management and treatment of HIDs and cancer.

## 1. Introduction

Although some heat shock proteins (HSPs) are constitutively produced, most are molecular chaperones that are normally over-expressed by cells in response to inducible signals that may lead to protein denaturation [1]. These stressors include heat, nutrient deficiency, oxidative stress, acute or chronic inflammatory diseases, viral infections, ischemia, heavy metals, exercise, gravity, and bacterial infections [2,3,4,5]. These responses enable cellular protection against protein denaturation and possible degradation of misfolded proteins, which may, in turn, result in protein aggregation and cancer [6]. Some of these constitutively-expressed heat shock polypeptides are involved in protein folding and translocation of organelles across cellular membranes, prompting many authors to label them “molecular chaperones” [5,6,7,8].

Since the discovery of heat shock proteins in 1962 by Ritossa in the salivary glands of the *Drosophila* larvae, the important functions of heat shock proteins in response to various stressful signals including human cancer and cystic fibrosis, has been well elucidated [9,10,11]. Upon discovering these proteins, it is not surprising that HSPs have made a very large impact in various areas of research, including medical and biological fields, because of their diverse functions in both pathological and normal conditions [12,13].

Molecular chaperones, which are found in all living cells and form part of the defence system against both internal and external stressors, are primarily grouped into two major groups according to their amino acid composition, molecular weight, as well as their specific cellular function as the high molecular weight and the small molecular weight HSPs [14,15]. The high molecular weight HSPs which range from 60 to 110 kDa are ATP-dependent and their primary cellular function is binding and folding of nascent proteins through ATP-dependent allosteric organization, even though assembling, transportation, vaccination against cancer metastasis, and degradation of improperly-folded peptides have also been reported [16,17]. Small molecular weight HSPs or heat shock protein β (HspBs), which range from 15 to 43 kDa, are ATP-independent molecular chaperones, of which their functions have been documented in embryo developmental processes, formation of respiratory organs, like cardiac muscles, as biomarkers for tumour formation, in exercise-induced stress, as well as in protein folding [5,15,18]. The classification, localization, and roles of HSPs are highlighted as shown (see Table 1).

The roles of HSPs in the pathology of many diseases including human inflammatory diseases (HIDs), has been well documented. Hsp70 and Hsp60 in particular have been reported to form part of the auto-antigen complex capable of eliciting immunoregulatory cascades, thus suppressing the immune response which is commonly observed in various HIDs, such as Type 1 diabetes, atherosclerosis, rheumatoid arthritis, asthma, and allergies [27]. Hsp functions in the immunology of HIDs could be attributed to their diverse properties: (1) their ability to assemble immune system apparatus to infectious sites; (2) they are capable of interacting with antigen-presenting cells and initiating CD8+ immune responses and are, therefore, seen as potential cancer vaccines; (3) their ability to refold denatured proteins, including most immune cells, thus promoting their survival under stressful conditions [28,29]. In this review, we discuss in detail the interplay between HSPs, apoptosis, reactive oxygen species (ROS) and inflammatory diseases, as well as possible roles and potential target HSPs hold in HIDs and cancer.

## 2. Role of HSPs in Apoptosis

Apoptosis, which is alternatively called programmed cell death or “cellular suicidal”, is a process by which cells are selectively killed without deteriorating neighbouring tissues [30]. This process is normally induced during embryo development, cell division, aging, as well as maintenance of cellular homeostasis, although it has also been reported to form part of the immune defence mechanism in response to cellular damage including the onset of cancer, and neurodegenerative and human inflammatory diseases (HIDs) [31,32,33]. Inappropriate stimulation of apoptosis has been associated with a variety of human diseases, such as ischemic damage, neurodegenerative disorders, autoimmune diseases, as well as cancer, making it a good therapeutic candidate against human diseases [32].

Studies have shown that apoptosis is mediated following the activation of caspases (a group of aspartate-specific cysteine proteases) that catalyses the addition or removal of specific cysteine or aspartic acid residues from target substrates, thereby activating or inhibiting the action of the targeted substrate [34,35]. These endoproteases are produced in an inactive state as zymogenes but upon activation, they play a central role in controlling apoptosis-mediated cell death, pyroptosis, necroptosis, as well as inflammatory reactions [36]. Caspases are broadly classified according to their roles in biological processes: apoptotic caspases (-7,-8, and -9) and inflammatory caspases (-1, -4, -5, and -12) in human and (-1, -11, and -12) in mice, respectively. The pro-inflammatory cytokines (such as interleukin 17, IL1β, TNF-α, IL-8, among others) and other inflammatory mediators are up-regulated as a result of the activation of caspases that are involved in inflammatory responses resulting in the innervation of innate immunity responses to cellular insults [36,37].

Caspase mediation of apoptosis falls into two broad categories: intrinsic and extrinsic mechanisms. Intrinsic or mitochondrial apoptotic pathway is a highly regulated and active pathway that cells use to antagonize mitochondrial stimulations as a result of stressors such as DNA damage, hypoxia, growth factor deprivation, as well as the accumulation of misfolded proteins. In response to cell death signals which activate the diverse functions of Bcl-2 families, intrinsic apoptosis is known to induce mitochondrial-membrane permeability through the opening of the permeability transition pore (PTP), thus allowing the release of cytochrome *c* (a pro-apoptotic factor that plays an important role in intrinsic apoptosis) into the cytosol. Inside the cytosol, cytochrome *c* complexes with apoptotic protease activating factor-1 (Apaf-)—an adaptor peptide—to recruit and activate pro-caspase-9, thereby forming a complex called apoptosome. The active caspase-9, on the other hand, triggers the activation and release of downstream ‘executioner’ caspase-3, which facilitates the degradation of the targeted substrate [34,36,38].

The extrinsic apoptosis pathway is characterised by interactions between Fas receptors (TNFR1, DR3 or death receptor 3, TRAIL-R1 or DR4, and Fas or CD95) and Fas ligands (TNF, Apo2-L, and FasL) on the surface of lymphocytes in response to suicidal signals. The binding of Fas ligand to FADD adaptor protein causes the dimerization of Fas associated death domains (FADDs) found in both Fas receptor and FADD adaptor proteins. This interaction allows the death effector domain (DED) to relate with pro-caspase-8 resulting in the formation of a complex called the death inducing signalling complex (DISC). The subsequent innervation of pro-caspase-8, which then triggers the activation of other pro-caspases, which then leads to the suicidal execution of cells [31,32,33,34,36,39,40].

Cells respond to numerous stressors, ranging from external to internal stressors, by expressing highly-regulated proteins upon thermal induction, called heat shock proteins (HSPs). These highly-conserved proteins are known for their diverse functions including protein folding, translocation of organelles across membranes, assembling and disassembling of proteins, signalling transduction, degradation of misfolded proteins, as well as ROS generation in the mitochondria capable of inducing apoptosis [41]. Overwhelming evidence has shown that HSPs have a wide array of functions in apoptosis, which in most cases, leads to the suppression of apoptotic pathways. Interestingly, the same stress signals that trigger apoptosis also stimulate the expression and release of HSPs. However, induction of HSPs represses apoptosis through inhibition of pro-apoptosis factors, such as p53, Bax, Bid, Akt, Apaf-1, and other Bcl-2 families. So far, numerous mechanisms of how HSPs incite cytoprotective effects against apoptosis has been proposed; one of them being the ability of Hsp27 to interact with cytochrome *c* and block its dimerization with Apaf-1, hence preventing the formation of apoptosome complex, which is the hallmark of mitochondrial cell suicide [36,42].

Work by Rane and colleagues have shown that Hsp27 relates directly with the serine/threonine (Akt) signalling pathway and this association inhibits neutrophil-mediated apoptosis in a phosphorylation-dependent manner [43]. Although the exact mechanism is still obscure, subsequent studies have shown that another molecular chaperone, Hsp70, directly associates with Apaf-1, blocking the production of apoptosome in an ATPase-dependent manner rather than its chaperoning activity [34,38,44]. Goto and co-workers and Beere reported that Hsp70 together with its co-chaperone Hsp40 homologs, inhibits nitric oxide mediated apoptosis by blocking the mitochondrial translocation of Bax, a pro-apoptotic member of Bcl-2 family in both ATPase and chaperoning dependent-fashion [34,45]. In spite of all the negative functions of HSPs in attenuating apoptosis, members of Hsp60 located in the mitochondria, in complexes with Hsp10, are involved in the signalling complex that results in pro-caspase-3 activation in cytochrome *c-*dependent apoptosis. In addition, several studies have shown that cytosolic Hsp60 associates with Bcl-2 proapoptic protein, Bax, leading to its activation, as well as its mediated apoptosis [46,47]. This observation suggests HSPs’ roles in apoptosis to be complex, complicated, and controversial [34,38].

## 3. HSPs and Oxidative Stress

Due to continuous mitochondrial oxidative respiratory reactions and other cellular and non-cellular processes, including phagocytosis, inflammatory reactions, ionizing radiation, air pollutants, exercise, cigarette smoking, and ozone [48,49,50], cells frequently generate reactive oxygen species (ROS) and reactive nitrogen species (RNS), which disturb normal oxidant and antioxidant cellular homeostasis, leading to oxidative stress [51,52]. These oxygen-containing compounds can be broadly categorized according to their oxygen-containing capacity: superoxide anion (O_2_^−^), hydroxyl radical (OH), alkoxyl radical (RO), peroxyl radical (HOO), nitric oxide radical (NO), nitrogen oxide (NO_2_), as well as potent non-radicals, such as hydrogen perioxide (H_2_O_2_), ozone (O_3_), and oxygen singlet (^1^O_2_) [53,54]. Both ROS and RNS in accumulated levels are very reactive and more potent than normal oxygen and nitrogen, thus causing deleterious effects to the living system.

In spite of all negativities associated with accumulated cellular ROS, several studies have shown that, at low or moderate levels of unknown concentration, ROS perform important cellular beneficial roles, including acting as secondary messengers in signal transduction, in immune defence, in antibacterial infections in the phagosome and vascular tone, as well as in ROS-induced programmed cell death in cancer cells [52,54,55,56,57,58]. The hydroxyl radical (OH^−^) which is the most reactive and most dangerous radical, is regenerated when H_2_O_2_ (a product of enzymatic reactions) decomposes slowly in the presence of Fe^2+^ in a process called the Fenton reaction and through other cellular reactions, including one between NO and O_2_^−^ to form a perioxynitrite intermediate which immediately decomposes to OH^−^ [59]. Upon OH^−^ formation, OH^−^ is capable of abducting electrons from biomolecules, especially lipids (polyunsaturated fatty acids), thereby inflicting DNA, carbohydrate, protein, and lipid oxidation and, most importantly, resulting in oxidative stress when the antioxidant system is supressed [58]. The body uses the antioxidant system to neutralise free radical cellular damage by converting them to less harmful substances under physiological conditions; however, this balance can be disrupted upon cellular stress leading to the accumulation of cellular levels of free radicals, especially ROS, thus activating many inflammatory cascades which have been implicated in various human inflammatory diseases (HIDs), such as arthritis, asthma, stroke, atherosclerosis, trauma, hepatitis, and in cancer [52,60,61,62].

The deteriorating cellular effects of free radicals in accumulated levels has been well documented; however, it is not surprising that biological systems protect themselves by increasing the expression level of highly-regulated proteins termed “heat shock proteins” in response to these reactive species (ROS) that may otherwise lead to oxidative stress. HSPs are known for their cytoprotective activities in response to a variety of cellular insults through their chaperoning activities ranging from polypeptide folding, assembling, and translocation of organelles across membranes, to conducting repairs, and the degradation of irreparable peptides [8,63]. Nevertheless, DNA fragmentation has been observed in cells undergoing ROS-mediated genotoxicity, but this effect has been rescued with the addition of the Hsp70 family, thereby suggesting that the cytoprotective effects of HSPs could be by protecting DNA breaks in response to ROS-induced insults [64]. Interestingly, HSPs have been reported to work hand-in-hand with the antioxidant system to inhibit or neutralise the cellular effects of ROS [65,66]. Accumulated ROS levels are said to induce apoptosis and are associated with a variety of inflammatory reactions, which is the hallmark of human inflammatory disease (HID) pathogenesis [53]. Hence, it is tempting to suggest that HSPs could play cytoprotective roles in the pathogenesis of HIDs and could be targeted as drug candidates for immunotherapy against HIDs.

## 4. HSPs in Human Inflammatory Diseases (HIDs) and Cancer

Inflammation, which forms part of the human first line of defence in response to stressful insults (such as pathogen invasion, oxidants, and cell damage), is characterized by swelling, pain, heat, and redness in the infected area [67]. This response enables cellular injury repair as well as elimination of any sign of necrotic cells, thereby activating innate immunity [68]. The inflammatory reaction is considered beneficial to humans in response to cellular insults because of the fact that it helps to clear and repair damaged cells and tissues. However, long-term unregulated inflammation may result in chronic inflammatory reactions marked with massive tissue and cell destruction, and this has been reported to play central role in the pathogenesis of many human inflammatory diseases (HIDs) [53]. HSPs have been reported to prevent inflammation through the inhibition of pro-inflammatory cytokines including tumour necrosis factor-α (TNF-α). The following sections in this review will hereby focus on the roles of HSPs in adult respiratory distress syndrome, rheumatoid arthritis, asthma, and cancer.

### 4.1. Acute Respiratory Distress Syndrome

Adult Respiratory Distress Syndrome (ARDS), which is alternatively called acute respiratory distress syndrome, is a lung inflammatory disorder characterized by Diffuse Alveolar Damage (DAD) as a result of the influx of liquid into the alveoli sacs (the site of blood-oxygenation), as well as the imbalance between pro-and-anti-inflammatory cytokines (interferon, TNF-α, interleukins, platelets derived growth factor) which, in most cases, leads to severe hypoxemia, stiffness of the lungs, pulmonary infiltration and organ failure, without causing cardiogenic pulmonary oedema [69,70]. Although ARDS mortality depends on several factors such as age, critical illness and other medical complications, ARDS has been estimated according statistical reports, to affect approximately 50 in every 100,000 people, resulting in almost 40% of deaths in infected patients worldwide [71,72]. So far, the actual causes of ARDS are obscured but, in most cases, ARDS has been reported in trauma or critically ill patients. Age and other unhealthy lifestyles, like smoking and chronic alcoholism, have also been documented as predisposing factors associated with ARDS cases [73].

In response to severe DAD cases, alveoli sac permeability of lung membranes is increased; this allows for the influx of neutrophils, tumour necrosis, macrophage inhibitory factor, together with platelet activation and sequestration, which are believed to be the centre stage for the development, progression, and pathogenesis of ARDS. However, elimination of activated inflammatory cytokines that cause tissue and cellular destructions in inflamed area have been reported to decrease morbidity and deaths in HIDs [74]. Nevertheless, heat shock proteins are known for their cytoprotective effects in response to cellular insults including inflammatory diseases; these proteins are said to be up-regulated during this stage of infection. Wesis and colleagues demonstrated that Hsp70 has the ability to suppress inflammatory responses by initiating the refolding of protein aggregates, thereby preventing the cellular damage and destruction observed in the pathology of ARDS and sepsis [75]. In support of this finding, other studies have shown that decreased mortality rates immediately after heat shock protein administration to endotoxin may mark the events of ARDS after several hours of development, as previously observed in rats [72,76]. Overwhelming evidence has shown that loss of pulmonary cells promote cell division which contribute massively to the pathogenesis of ARDS and heat shock proteins, Hsp70 in particular, has been previously shown to limit inflammation in HIDs, by inhibiting the pathway that leads to nuclear factor (NF)-kB activation as observed in pneumocytes [75]. Although the mechanism of Hsp-cytoprotective action in ARDS is largely unknown, it has been reported to follow the same mechanism as previously observed in other lung inflammatory diseases, like sepsis and pneumocystis [77].

### 4.2. Rheumatoid Arthritis

Rheumatoid arthritis (RA) is a long-term autoimmune-inflammatory disease where, instead of the immune system defending the body, it attacks synovial fluid-membranes normally found in the wrist, hand, or joints of the feet. RA, like other inflammatory diseases, is characterised by stiffness, swelling, and warmness and pain of the joints which, if left untreated long-term, may result in severe inflammation, deformity, and several functional disabilities [78,79]. Severe inflammation is said to attract numerous immune cytokines, chemokine, lymphocytes, and other immune components to the area of the infection (normally in the joints), causing redness, warmness, and painful discomfort, which are the symptoms, observed in rheumatoid arthritis infection. To date, the exact cause of RA is poorly understood and there is no pronounced cure. However, RA has been reported to arise as a result of family history or genetics (people that are genetically predisposed to RA) and other predisposing factors, such as environmental effects, educational background, and low socio-economic status, as well as unhealthy lifestyles, such as smoking and lack of exercise [78,80,81,82].

In spite of all the efforts in treatment, different medications and improvement in lifestyle with added exercise and healthier nutrition, RA remains one of the leading inflammatory autoimmune diseases worldwide. According to statistical reports, 24.5 million people were effected by RA in 2015, with a rate of 100,000 people every year [78]. RA, which occurs more in middle-aged females than in males, and has shown 10% mortality increase between 1990 and 2013, making it one of the prevalent health concerns according to the National Institute of Health (NIH).

In response to RA infection which is characterized by severe inflammation, biological systems always increase the synthesis of heat shock proteins especially Hsp70, the most inducible protein upon stress. Hsp70 exerts its anti-apoptotic activities by inhibiting pro/inflammatory signals or factors that lead to apoptosis, inflammatory pathways, such as activation of caspases, JNK (Jun N-terminal) signalling pathway, the release of cytochrome *c*, and the formation of apoptosome, which is the hallmark of apoptosis and inflammation progression [34]. More so, it is not surprising that Hsp70 is, therefore, up-regulated in the synovial membrane during rheumatoid arthritis fibroblast-like synoviocyte (RA-FLSs) infection to modulate the effects of T-cells, as well as to control inflammation via inhibition of pro-inflammation signals [83]. Surprisingly, Kang and colleagues reported that repression of Hsp70 by siRNA in an in vitro experiment decreased inflammation by protecting RA-FLSs from nitric oxide mediated-programmed cell death, although the actual function of Hsp70 in the RA in vivo experiment is still not yet clear [84]. This observation suggests the pro-apoptotic and negative roles of Hsp70 in the pathogenesis of RA FLSs infection; thus, inhibition of Hsp70 expression in RA could be one of the mechanisms of controlling severe inflammation observed in rheumatoid arthritis. In addition, van Room and co-workers showed that T-cells taken from RA patients were able to react with human or self-Hsp60 and inhibit the activation of TNF-α (a pro-inflammatory factor) through the activation of Th2 cytokine regulator, whereas there were no regulatory effects observed in Hsp65 isolated from *Mycobacterium tuberculosis* [85,86]. Consistent with this finding, several studies have shown that T-cell response to self-Hsp70 and Hsp60 through production of interleukin-4 and interleukin-10 regulatory cytokines suppresses arthritis diseases in many animal models. These observations suggest that cross-reactivity of HSPs with T-cells could be one of the ways of controlling human inflammatory diseases, like RA, under stressed physiological states [86,87]. Taken together, the self-Hsp60 reactivity observed in Lewis rats and adjuvant arthritis [88] makes it easy to speculate that human Hsp60 and mycobacterial Hsp60 could be used as potential vaccines against autoimmune inflammatory diseases since they are capable of eliciting immune responses.

### 4.3. Asthma

Asthma is a multigenic and multifactorial bronchial chronic inflammatory disorder characterised by airway obstruction, bronchospasm and remodelling of the bronchial wall, thus resulting in thickness of airflow walls and difficulties in breathing [89]. The symptoms of asthma, which can be caused by genetic or environmental factors (allergens and air pollutions), or both, range from chest tightness and wheezing sounds when breathing, down to shortness of breath; all these symptoms may vary in individuals. The various associations of different cellular networks, such as smooth muscle, macrophages, fibroblasts, eosinophil and epithelial cells may result in airway remodelling and inflammation [90]. This process of remodelling promotes further thickening of bronchial walls and narrowing the airflow, leading to the breathing difficulties commonly observed in the pathogenesis of asthmatic patients [91].

Statistics have shown that asthma affects 358 million people worldwide which has, so far, resulted in 397,100 deaths in 2015 alone, as compared to the 183 million people who were affected in 1990 [92,93]. According to the Global Initiative for Asthma (GINA), South Africa remains the fourth leading country, globally, and the first on the African continent with the highest mortality resulting from asthma attacks [94]. Furthermore, an estimated 3.9 million South Africans were affected by asthma in 2012, accounting for 1.5% of deaths in this country every year [95]. Taking into account the cytoprotective roles of HSPs in response to cellular insults, several authors have reported the over-expression of Hsp70 in asthmatic patients [96,97]. Surprisingly, recent studies have suggested that intracellular synthesis of Hsp70 chaperones in airways and alveolar sacs of asthma patients correlate with the deleterious effects and severity of the disease, probably by forming part of the inflammatory complex, where it may up-regulate THP-1 synthesis by inducing CD23 expression in the Th2 environment [98,99,100]. Nevertheless, initial studies have proposed its autoprotective activities in response to asthma and lung complications by inhibiting TNF-α mediated-inflammation [96,101]. In support of this finding, many studies have conclusively stated with evidence that the interaction between anti-Hsp70 and anti-Hsp60 correlates with progression, poor prognosis, and severity observed in asthmatic patients, as previously suggested by Shingai and colleagues in a patient with autoimmune liver disease [102] and *Mycobacterium* Hsp65 in chronic human atherosclerosis [103]. Interestingly, serum Hsp70 circulation is said to increase in pregnant asthmatic patients and this elevated Hsp70 level correlates with foetal and maternal complications such as low birth weight in infants, pre-eclampsia and preterm delivery, and these result in an almost 35% increased death rate in asthmatic pregnant women [104]. Other factors such as smoking and maternal obesity, on the other hand, have also been reported to promote perinatal death [105].

The exact mechanism of Hsp70 activity during asthma development remains to be seen, although evidence has it that Hsp70 mostly targets T-cells and humoral immunity in response to infectious agents, and this may provide a link between T-lymphocyte cross-reactivity-induced autoimmunity and immune responses to infectious diseases [106]. Another possible explanation is the ability of Hsp70 to interact with antigen presenting cells (APCs) and amplify its activities, which plays a crucial role in the modulation and initiation of asthmatic attacks in chronic asthma patients [96,104]. Additional studies have shown the up-regulation of Hsp90 and Hsp72 in response to ROS-induced asthma attacks in young children. Even though the exact functions of Hsp90 and Hsp72 remain unknown, it has been suggested that the elevated levels of these heat shock proteins could be to refold denatured proteins that may result from ROS induced-oxidative stress [107], thereby preventing protein aggregates, cellular deteriorations, and further complications. Tong and Luo showed that elevated Hsp70 levels in peripheral blood mononuclear cells (PBMCs) suggest Hsp involvement in the pathogenesis of asthma infection [108]. Through these observations we, therefore, propose that enhancing Hsp inhibitors in asthmatic patients could reduce the severity in this disease and will become another interesting aspect in the management of asthma and other lung inflammatory diseases, nonetheless more research is needed in this area of study.

### 4.4. Cancer

Cancer is a term given to the group of diseases characterized by the abnormal invasion and proliferation of cells as a result of uncontrolled cell divisions or mutated tumour suppressor genes [109]. Cancer remains one of the major sources of global morbidity and mortality and persists as the leading cause of death in individuals below 85 years old in the United States despite recent improvements in treatment modalities [53,110]. This exponential increase in cancer cases could be due to increasing prevalence in related factors, such as obesity, unhealthy life styles (smoking, lack of physical activity and imbalance diet), as well as genetic predisposition [31].

Hanahan and Weiberg proposed that the mechanisms by which cancerous cells survive in the human body are by increased resistance to anti-proliferative signals and apoptosis induced-suicidal or abnormal cell death [111]. Interestingly, several studies have reported that this resistance to apoptosis and antiproliferative signals observed in cancerous cells are actually induced in the presence of HSPs, which favour the refolding of denatured peptides through Hop (Hsp70/Hsp90 organizing protein) chaperoning related activities instead of peptide degradation [112,113]. Previous studies have shown that HSPs inhibit both pro-inflammatory and pro-apoptotic caspases, by binding and blocking their activation, thus increasing cancer cell survival [114]. Overexpression of Hsp27 in prostrate, ovarian and bladder cancers have been shown to correlate with down-regulation of p53 induced-stimulation of the p21 gene and up-regulation of matrix metalloproteins (MMPs), proteins that are known to pave the way for cancer cell migration, invasion, proliferation, and metastasis, thereby inhibiting p53 mediated senescence, apoptosis and cell cycle arrest [115]. These observations are simple indications of the roles of Hsp27 in prognosis, angiogenesis, proliferation, as well as metastasis of cancer cells.

In line with this, Hsp27 has been reported to promote epithelial-to-mesenchymal transition (EMT) in prostate cancer patients. EMT is an important event that occurs during organ morphogenesis and embryogenesis, characterized by the actin cytoskeleton remodelling, loss of apico-basolateral polarity and cell-cell junction dissolving, thus resulting in proliferation and mobility of cancer cells [116]. Down-regulation of Hsp27 using OGX-427 (an antisense therapy) decreases cancer cell invasion, migration, phosphorylation of LL-6-dependent STAT3 (a major mediator of EMP in many cancer types) and nuclear translocation, as well as matrix metalloproteinase, thereby reversing EMT activity. This observation suggests that Hsp27 is needed for interleukin LL-6 to induce EMP possibly through the modulation of the STAT3 signalling pathway [117].

Similarly, overexpression of Hsp70 corresponds with an increased proliferation of malignant cells and knockdown of Hsp70 experiments in various malignant tumours have been shown to increase the susceptibility of cancer cells to certain chemotherapy agents, suggesting the negative roles of Hsp70 in the invasiveness and proliferation of cancerous cells [118]. In addition, Hsp70 also acts as a cancerous cell-surviving factor by inhibiting TNF-α mediated-apoptotic cell death, thus promoting carcinogenesis and cancer cell oncogenic potential through the mechanism of escape immunology [115]. Furthermore, studies have reported that either Hsp90 or Hsp70 can bind and block the activation of apoptotic protease activating factor 1 (Apaf-1) and indirectly inhibit pro-caspase activation, apoptosis, as well as enhance abnormal cell survival [119]. Nevertheless, Hsp60 has been reported to show pro-carcinogenic activity through the inhibition of dusterin in neuroblastoma cells, cyclophilin D mediated-mitochondrial cell death, and promotion of cell survival via nuclear factor-kB activation [120,121].

Conversely, previous in vivo experiments by Chalmin and colleagues showed that the down-regulation of Hsp70 correlates with cancer cell survival due to the reduced immune killing of cancer cells [122]. Consistent with this finding is that Hsp40 homolog-DNAJA3 has been shown to inhibit squamous cell carcinoma invasion, migration, growth, recurrence and proliferation in both in vivo and in vitro experiments [115], possibly through induction of mitochondrial apoptosis, as previously observed in MCF-7 breast cancer cells [123]. These findings suggest that Hsp70 could suppress tumour cells when co-expressed with DNAJA3 in the absence of other molecular chaperones, like Hsp90, via CHIP (C-terminal HSP70 interacting protein)-mediated protein degradation.

## 5. Association between Heat Shock Proteins, Oxidative Stress, Apoptosis, Human Inflammatory Diseases (HIDs), and Cancer

Although accumulated levels of ROS have been proposed to cause deleterious effects to biomolecules, such as lipids, carbohydrates, proteins, and nucleic acids, ROS at moderate levels of unknown concentrations have been documented to play significant roles, such as acting as secondary messengers in signal transduction, immune defence, antibacterial infections in the phagosome, as well as ROS-induced programmed cell death in cancer cells [52,54]. Biological systems employ antioxidants (reduced glutathione, catalase, superoxide dismutase) to nullify the negative effects of free radicals, as well as to prevent ROS-mediated cellular damages and functional impairments. However, this antioxidant system can be supressed under severe cellular stress conditions as a result of elevated ROS levels, resulting in oxidative stress and amplification of inflammatory reactions, as well as activation of immune system cascades, a proposed central stage in progression and pathogenesis of several HIDs [53,124,125].

Inflammation is said to be part of the first line of the innate immunity complex that responds to cellular damage caused by infectious agents or xenobiotics [53]. Onset of stress has been established to attract several inflammatory cells, such as cytokines, macrophages, and chemokines, at the site of damaged cells, which is a process mediated upon toll-like receptor (TLRs) activation. The aim of this TLR mediated-inflammatory response is to eliminate detrimental cells and promote cellular repair via the mechanism of apoptosis. However, long-term un-regulated inflammatory reaction may lead to excessive and amplified apoptosis, resulting in chronic inflammation characterized by massive tissue and cellular destruction, commonly seen in many chronic and neurodegenerative diseases [62,126].

The roles of ROS induced-apoptosis in inflammatory reactions can be viewed as a double-edged sword. ROS-induced apoptosis under normal cellular conditions performs beneficial roles in suicidal killing of cells via mitochondrial apoptosis, but the abnormal stimulation of this mechanism under stressful conditions could result in excessively amplified intrinsic apoptosis, leading to the massive cellular destructions observed in the aetiology of several HIDs [127,128]. In line with this, elevated levels of ROS in the airways of asthmatic and ARDS patients suggests their roles in the pathogenesis and progression of HIDs by inducing severe inflammation [129,130]. HSPs, on the other hand, are mostly induced upon heat stress and, therefore, it is not surprising that HSPs are highly expressed in the inflamed area, possibly to refold denatured peptides caused by ROS-induced reperfusion injury on the inflammation sites identified in rheumatoid arthritis [131]. Heat is also one of the major characteristics of inflammation, and excessive inflammation leads to HIDs. Although some HSPs perform pro-inflammatory/apoptotic functions, most HSPs are known for their anti-apoptotic/inflammatory potential, including the inhibition of pro-inflammatory/apoptotic factors or pathway capabilities such as through the nuclear factor (NF-kB), activation of caspases and c-Jun NH2-terminal kinase pathway [132]. Suppression of HSP expression levels, therefore, leads to worse inflammation cases, which can be linked to the severe inflammation seen in several HIDs.

## 6. Future Direction in the Use of HSPs as Therapeutic Candidates

From this point, it can be proposed that carefully regulation of inflammatory responses, induction of apoptosis and endogenous generation of ROS would definitely help in the management or treatment of HIDs. In many studies, HSPs have been reported to play crucial roles in the pathogenesis of HIDs and cancer due to their modulating effects in inflammation cascades that lead to the endogenous generation of ROS and apoptosis [27,34,133], possibly via their chaperoning activities of refolding misfolded proteins, or via inhibition of pro-inflammatory cytokines under pathological conditions. Under stressful conditions, HSPs has been suggested to play a prominent role by binding to the lipid rafts inside lipid membranes, thus maintaining lipid membrane stability, physical orderliness, as well as preventing lipid membrane functional impairments. Altered membrane functionality has been associated with cancer, neurodegenerative diseases, and diabetes, suggesting the possible role of HSPs as therapeutic targets in the management of these diseases [134].

Up–regulation of HSPs in cancerous cells has been well documented and has been associated with poor prognosis, proliferation, cell differentiation, invasion, progression, and metastasis [135,136]. Among others, Hsp90, Hsp70, and Hsp27 in particular, have been reported by many studies to increase tumour cell survival via inhibition of pro-inflammatory cytokines and ROS-mediated apoptosis [119,136,137]. Chauhan and co-workers demonstrated that Hsp27 can promote the survival of malignant tumours by conferring resistance to the inflammatory drug dexamethasone (a drug for treating HIDs such as rheumatoid arthritis, skin inflammation, and cancer) in myeloma cell lines via the inhibition of SMAC (mitochondrial release of second mitochondrial-derived activator of caspases) and cytochrome *c*, both of which are masters of intrinsic apoptosis mediators [138].

Hsp27 can also act as an anti-apoptotic factor by promoting the activities of nuclear factor-kB (NF-kB) while blocking apoptosis pathways mediated by NF-kB inhibitor (IkBα), thereby promoting cancer cell proliferation and survival [135]. Hsp27 involvement in cancer could be through phosphorylation at three-serine residues mediated by MAPKAPK2 (mitogen-activated protein kinase activated protein kinase). This phosphorylation enables the Hsp27 to form oligomers up to 100 kDa, making it ideal in preventing protein aggregation by refolding denatured peptides in an ATP-independent manner [119]. Similarly, Hsp70 and Hsp90 function in cancer cell survival has been previously elucidated. Work by Nylandsted and colleagues showed that selective inhibition of Hsp70 in breast cancer lines increased the susceptibility of cancer to chemotherapy and sensitized them to caspase-mediated apoptotic death [139]. Hsp90 is known to play a crucial role in cancer cell survival and has been reported as a drug target in many cancer types. Inhibition of Hsp90 in leukaemia, colorectal, breast, lung, melanoma, and bladder cancer correlates with decreased invasion, motility, and prognosis of cancer, as well as an increase in the susceptibility of cancer cells to therapy [115]. This may be possible via the inhibition of signalling pathways that confer resistance to chemotherapy. In view of this, Zuninga and Shonhai previously postulated that Hsp70 and Hsp90 are the most druggable HSPs due to the fact that most Hsp inhibitors either mimic or target their ATPase activity [140]. This could be via the inhibition of Hsp70/Hsp90 organizing protein (Hop), which favours the refolding of aberrant peptides while blocking CHIP-mediated peptide degradation. Interestingly, subset studies have shown that physical exercise induces Hsp expression [141,142,143], and is one of the ways of managing HIDs and cancer. Furthermore, one can then speculate that one of the health benefits of exercise is to induce Hsp expression, which is known for its cellular cytoprotective activities, probably by forming part of the immune protective system against infection.

**Figure 1:**
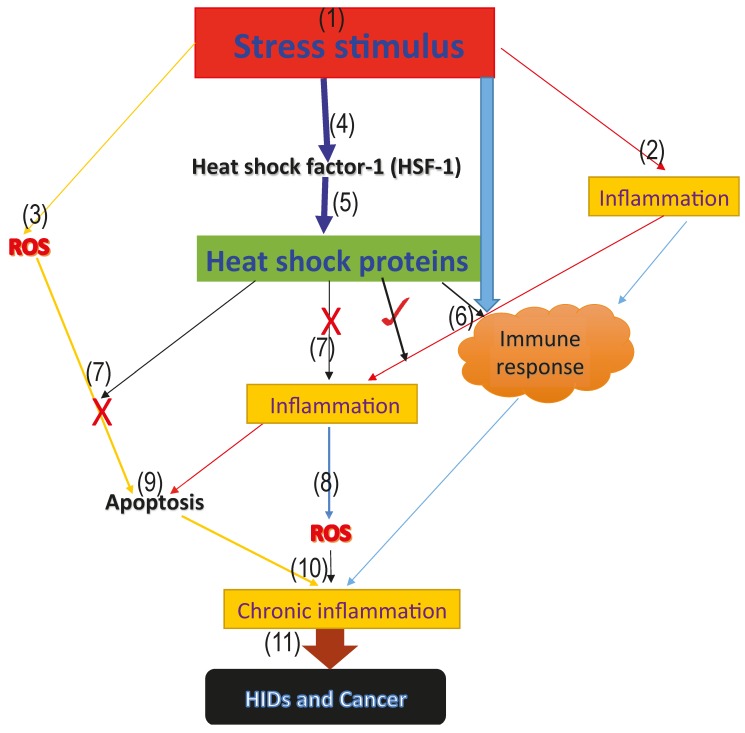
Model proposing the roles of heat shock proteins in HIDs and cancer. The above model represents the proposed roles that heat shock proteins play in human inflammatory diseases and cancer. (1) the onset of the stress signal; (2) stress activates inflammatory reactions which aims at repairing the damage caused by the stress; (3) generation of ROS from the infected area; (4) activation of the heat shock factor-1 (HSF-1), which increases the synthesis of the cytoprotective heat shock proteins; (5) activation of heat shock proteins; (6) stress, as well as inflammation and heat shock proteins, activate the immune response and form part of the innate immune response (7); cytoprotective heat shock proteins inhibit further generation of ROS, as well as inflammation, thus blocking excessive ROS and inflammation mediated-apoptosis via the inhibition of pro-inflammatory and pro-apoptotic factors; (9) excessive apoptosis mediated by ROS; (8) accumulated level of ROS leading to oxidative stress; (10) immune response, inflammatory reaction, accumulated ROS level and excessive apoptosis mediated by ROS as a result of antioxidant suppression, leading to oxidative stress and chronic inflammation marked with massive cellular and tissue destruction; and (11) long-term uncontrolled chronic inflammation degenerates to HIDs and cancer.

The onset of stress increases ROS generation, as well as inflammatory reactions. The generated ROS and inflammation activates the immune response and induces apoptosis, which aims at fighting off the infectious agent. However, if this mechanism is not well stimulated, it could degenerate to chronic inflammation. Interestingly, heat shock factor-1 (HSF-1) is also upregulated during this stage of infection. HSF-1 increases the synthesis of protective HSPs, which stops inflammatory reactions and massive cellular destruction through apoptosis, as well as further ROS generation, possibly via inhibition of pro-inflammatory factors and activation of the immune system, thus preventing chronic inflammation, HIDs, and cancer progression, as proposed in Figure 1.

HSPs have been reported to perform beneficial cytoprotective effects when induced prior to inflammation and deleterious effects after propagation of pro-inflammation reactions [132]. We, therefore, proposed that induction of HSPs prior to inflammation and carefully regulation of ROS, inflammation and apoptosis through the induction of HSPs as well as the inhibition of HSPs in cancer and certain HIDs (asthma and ARDS) and enhancement of HSP activities in RA may, and will, serve as future study references as proposed in the model (Figure 1), which highlights the possible roles of HSPs in HIDs and cancer.

## 7. Conclusions

The search for new drugs for the treatment of HIDs and cancer continues, and new studies are now focused on discovering drugs that will have minimal side effects. Recently, HSPs have attracted a great deal of research interest because of their ever-present occurrence in a variety of human diseases, including HID-tested patients, even though their action in some HIDs is still unclear. From our perspective as proposed in the model (Figure 1). We, therefore, suggest that targeting HSPs in HIDs will serve as good potential candidates towards the treatment and management of many HIDs, as well as early detection of these diseases.

## Figures and Tables

**Table 1:** Classification of heat shock protein families.

Classification	Location	Cellular Function	Reference
Hsp10	Mitochondria	Serves as biomarker in endometrial cancer and helps protein folding	[19,20]
Hsp27	Cytosol, endoplasmic reticulum & nucleus	Facilitates refolding of denatured proteins (chaperoning activity) and serves as a biomarker in many cellular diseases such as cancer	[21]
Hsp40	cytosol	Assists HSP70 in protein folding (co-chaperoning with HSP70)	[22]
HSP60	Cytoplasm & mitochondria	Assists in protein folding, prevents protein aggregation and assembling of unfolding proteins via the formation of the hetero-oligomeric complex	[23]
Hsp70	Cytoplasm & nucleus	Aids protein assembling, protein folding, degradation of improperly folded peptides and translocation of organelles	[5]
Hsp90	Cytoplasm	Assists in protein folding, refolding and degradation. It also facilitates signal transduction and important roles in cancer and sarcomere formation as well as in myosin folding	[5,24]
Hsp100	Cytoplasm	Complexes with other HSPs to refold aggregated or misfolded proteins	[25]
Hsp110	Cytosol & nucleus	Helps immune response and complexes with HSP70 to promote protein refolding and cell survival under stress	[26]

## References

[1] Srivastava P. (2002). Roles of heat-shock proteins in innate and adaptive immunity. Nat. Rev. Immunol..

[2] Asea A. (2003). Chaperokine-induced signal transduction pathways. Exerc. Immunol. Rev..

[3] Searle S., McCrossan M.V., Smith D.F. (1993). Expression of a mitochondrial stress protein in the protozoan parasite Leishmania major. J. Cell Sci..

[4] Zügel U., Kaufmann E. (1999). Role of Heat Shock Proteins in Protection from and Pathogenesis of Infectious Diseases. Clin. Microbiol. Rev..

[5] Jee H. (2016). Size dependent classification of heat shock proteins: A mini-review. J. Exerc. Rehabil..

[6] Sharma G., Nath A., Prasad S., Singh N., Dubey P., Saikumar G. (2012). Expression and Characterization of Constitutive Heat Shock Proteins 70.1 (HSPA-1A) Gene in In Vitro produced and In Vivo-Derived Buffalo (Bubalis) Embryos. Reprod. Domest. Anim..

[7] De Maio A. (1999). Heat shock proteins: Facts, thoughts, and dreams. Shock.

[8] Shiber A., Ravid T. (2014). Chaperoning proteins for destruction: Diverse roles of HSP70 chaperones and their co-chaperones in targeting misfolded proteins to the proteasome. Biomolecules.

[9] Daugaard M., Kirkegaard-Sørensen T., Ostenfeld M.S., Aaboe M., Høyer-Hansen M., Ørntoft T.F., Rohde M., Jäättelä M. (2007). Lens epithelium-derived growth factor is an HSP70-2 regulated guardian of lysosomal stability in human cancer. Cancer Res..

[10] Singh O.V., Pollard H.B., Zeitlin P.L. (2008). Chemical rescue of ΔF508-CFTR mimics genetic repair in cystic fibrosis bronchial epithelial cells. Mol. Cell. Proteom..

[11] Zhang H., Liu R., Huang W.A. (2007). 14-mer peptide from HSP70 protein is the critical epitope which enhances NK activity against tumor cells in vivo. Immunol. Investig..

[12] De Maio A. (2011). Extracellular heat shock proteins, cellular export vesicles, and the Stress Observation System: A form of communication during injury, infection, and cell damage. Cell Stress Chaperones.

[13] De Maio A., Santoro M.G., Tanguay R.M., Hightower L.E. (2012). Ferruccio Ritossa’s scientific legacy 50 years after his discovery of the heat shock response: A new view of biology, a new society, and a new journal. Cell Stress Chaperones.

[14] Garbuz D.G., Astakhova L.N., Zatsepina O.G., Arkhipova I.R., Nudler E., Evgen’ev M.B. (2011). Functional organization of *hsp70* cluster in camel (*Camelus dromedarius*) and other mammals. PLoS ONE.

[15] Lanneau D., Wettstein G., Bonniaud P., Garrido C. (2010). Heat Shock Proteins: Cell Protection through Protein Triage. Sci. World J..

[16] Sung Y.Y., MacRae T.H. (2011). Heat shock proteins and disease control in aquatic organisms. J. Aquac. Res. Dev. S.

[17] Wan T., Zhou X., Chen G., An H., Chen T., Zhang W., Liu S., Jiang Y., Yang F., Wu Y. (2004). Novel heat shock protein HSP70L1 activates dendritic cells and acts as a Th1 polarizing adjuvant. Blood.

[18] Juo L.Y., Liao W.C., Shih Y.L., Yang B.Y., Liu A.B., Yan Y.T. (2016). HSPB7 interacts with dimerized FLNC and its absence results in progressive myopathy in skeletal muscles. J. Cell Sci..

[19] Dubé V., Grigull J., DeSouza L.V., Ghanny S., Colgan T.J., Romaschin A.D., Siu K.M. (2007). Verification of endometrial tissue biomarkers previously discovered using mass spectrometry-based proteomics by means of immunohistochemistry in a tissue microarray format. J. Proteom. Res..

[20] Meyer A.S., Gillespie J.R., Walther D., Millet I.S., Doniach S., Frydman J. (2003). Closing the folding chamber of the eukaryotic chaperonin requires the transition state of ATP hydrolysis. Cell.

[21] Vidyasagar A., Wilson N.A., Djamali A. (2012). Heat shock protein 27 (HSP27): Biomarker of disease and therapeutic target. Fibrogenesis Tissue Repair.

[22] Li J., Qian X., Sha B. (2009). Heat shock protein 40: Structural studies and their functional implications. Protein Pept. Lett..

[23] Belles C., Kuhl A., Nosheny R., Carding S.R. (1999). Plasma membrane expression of heat shock protein 60 in vivo in response to infection. Infect. Immun..

[24] Tuttle J.A., Castle P.C., Metcalfe A.J., Midgley A.W., Taylor L., Lewis M.P. (2015). Downhill running and exercise in hot environments increase leukocyte Hsp72 (HSPA1A) and Hsp90α (HSPC1) gene transcripts. J. Appl. Physiol..

[25] Krobitsch S., Brandau S., Hoyer C., Schmetz C., Hübel A., Clos J. (1998). Leishmania donovani heat shock protein 100 characterization and function in amastigote stage differentiation. J. Biol. Chem..

[26] Zuo D., Subjeck J., Wang X.Y. (2016). Unfolding the role of large heat shock proteins: New insights and therapeutic implications. Front. Immunol..

[27] Van Eden W., Van der Zee R., Prakken B. (2005). Heat-shock proteins induce T-cell regulation of chronic inflammation. Nat. Rev. Immunol..

[28] Wu Y., Wan T., Zhou X., Wang B., Yang F., Li N., Chen G., Dai S., Liu S., Zhang M. (2005). Hsp70-like Protein 1 fusion protein enhances induction of carcinoembryonic antigen–specific CD8+ CTL response by dendritic cell vaccine. Cancer Res..

[29] Colaco C.A., Bailey C.R., Walker K.B., Keeble J. (2013). Heat shock proteins: Stimulators of innate and acquired immunity. BioMed. Res. Int..

[30] Ahmad R., Rasheed Z., Ahsan H. (2009). Biochemical and cellular toxicology of peroxynitrite: Implications in cell death and autoimmune phenomenon. Immunopharmacol. Immunotoxicol..

[31] Wong R.S. (2011). Apoptosis in cancer: From pathogenesis to treatment. J. Exp. Clin. Cancer Res..

[32] Elmore S. (2007). Apoptosis: A review of programmed cell death. Toxicol. Pathol..

[33] Lowe S.W., Lin A.W. (2000). Apoptosis in cancer. Carcinogenesis.

[34] Beere H.M. (2004). The stress of dying’: The role of heat shock proteins in the regulation of apoptosis. J. Cell Sci..

[35] Wolf B.B., Green D.R. (1999). Suicidal tendencies: Apoptotic cell death by caspase family proteinases. J. Biol. Chem..

[36] McIlwain D.R., Berger T., Mak T.W. (2013). Caspase functions in cell death and disease. Cold Spring Harb. Perspect. Biol..

[37] Novara G., Galfano A., Berto R.B., Ficarra V., Navarrete R.V., Artibani W. (2006). Inflammation, apoptosis, and BPH: What is the evidence?. Eur. Urol. Suppl..

[38] Takayama S., Reed J.C., Homma S. (2003). Heat-shock proteins as regulators of apoptosis. Oncogene.

[39] Jolly C., Morimoto R.I. (2000). Role of the heat shock response and molecular chaperones in oncogenesis and cell death. J. Natl. Cancer Inst..

[40] Leung A.M., Redlak M.J., Miller T.A. (2015). Role of heat shock proteins in oxygen radical–induced gastric apoptosis. J. Surg. Res..

[41] Bukau B., Weissman J., Horwich A. (2006). Molecular chaperones and protein quality control. Cell.

[42] Garrido C., Bruey J.M., Fromentin A., Hammann A., Arrigo A.P., Solary E. (1999). HSP27 inhibits cytochrome c-dependent activation of procaspase-9. FASEB J..

[43] Rane M.J., Pan Y., Singh S., Powell D.W., Wu R., Cummins T., Chen Q., McLeish K.R., Klein J.B. (2003). Heat shock protein 27 controls apoptosis by regulating Akt activation. J. Biol. Chem..

[44] Ravagnan L., Gurbuxani S., Susin S.A., Maisse C., Daugas E., Zamzami N., Mak T., Jäättelä M., Penninger J.M., Garrido C. (2001). Heat-shock protein 70 antagonizes apoptosis-inducing factor. Nat. Cell Biol..

[45] Goto H., Yano S., Matsumori Y., Ogawa H., Blakey D.C., Sone S. (2004). Sensitization of tumor-associated endothelial cell apoptosis by the novel vascular-targeting agent ZD6126 in combination with cisplatin. Clin. Cancer Res..

[46] Samali A., Cai J., Zhivotovsky B., Jones D.P., Orrenius S. (1999). Presence of a pre-apoptotic complex of pro-caspase-3, HSP60 and Hsp10 in the mitochondrial fraction of Jurkat cells. EMBO J..

[47] Gupta S., Knowlton A.A. (2002). Cytosolic heat shock protein 60, hypoxia, and apoptosis. Circulation.

[48] Lobo V., Patil A., Phatak A., Chandra N. (2010). Free radicals, antioxidants and functional foods: Impact on human health. Pharmacogn. Rev..

[49] Ji L. (1995). Oxidative stress during exercise: Implication of antioxidant nutrients. Free Radic. Biol. Med..

[50] Fubini B., Hubbard A. (2003). Reactive oxygen species (ROS) and reactive nitrogen species (RNS) generation by silica in inflammation and fibrosis. Free Radic. Biol. Med..

[51] Uttara B., Singh A.V., Zamboni P., Mahajan R.T. (2009). Oxidative stress and neurodegenerative diseases: A review of upstream and downstream antioxidant therapeutic options. Curr. Neuropharmacol..

[52] Alfadda A.A., Sallam R.M. (2012). Reactive oxygen species in health and disease. BioMed. Res. Int..

[53] Oyinloye B.E., Adenowo A.F., Kappo A.P. (2015). Reactive oxygen species, apoptosis, antimicrobial peptides and human inflammatory diseases. Pharmaceuticals.

[54] Lushchak V.I. (2014). Free radicals, reactive oxygen species, oxidative stress and its classification. Chem.-Biol. Int..

[55] Finkel T. (2011). Signal transduction by reactive oxygen species. J. Cell. Biol..

[56] Gonzalez C., Sanz-Alfayate G., Agapito M.T., Gomez-Nino A., Rocher A., Obeso A. (2002). Significance of ROS in oxygen sensing in cell systems with sensitivity to physiological hypoxia. Respir. Physiol. Neurobiol..

[57] Scandalios J.G. (2002). Oxidative stress responses-what have genome-scale studies taught us?. Genome Biol..

[58] Mittler R., Vanderauwera S., Suzuki N., Miller G., Tognetti V.B., Vandepoele K., Gollery M., Shulaev V., Van Breusegem F. (2011). ROS signaling: The new wave?. Trends Plant Sci..

[59] Coyle J.T., Puttfarcken P. (1993). Oxidative stress, glutamate, and neurodegenerative disorders. Science.

[60] Schneeberger K., Czirják G.Á., Voigt C.C. (2013). Inflammatory challenge increases measures of oxidative stress in a free-ranging, long-lived mammal. J. Exp. Biol..

[61] Lee J., Koo N., Min D.B. (2004). Reactive oxygen species, aging, and antioxidative nutraceuticals. Compr. Rev. Food Sci. Food Saf..

[62] Hsieh H.L., Yang C.M. (2013). Role of redox signaling in neuroinflammation and neurodegenerative diseases. BioMed. Res. Int..

[63] Mayer M.P., Bukau B. (2005). Hsp70 chaperones: Cellular functions and molecular mechanism. Cell. Mol. Life Sci..

[64] Jacquier-Sarlin M.R., Fuller K., Dinh-Xuan A.T., Richard M.J., Polla B.S. (1994). Protective effects of HSP70 in inflammation. Cell. Mol. Life Sci..

[65] Trott A., West J.D., Klaić L., Westerheide S.D., Silverman R.B., Morimoto R.I., Morano K.A. (2008). Activation of heat shock and antioxidant responses by the natural product celastrol: Transcriptional signatures of a thiol-targeted molecule. Mol. Biol. Cell..

[66] Wu C.-W., Biggar K.K., Zhang J., Tessier S.N., Pifferi F., Perret M., Storey K.B. (2015). Induction of antioxidant and heat shock protein responses during torpor in the gray mouse lemur, Microcebus murinus. Genom. Proteom. Bioinform..

[67] Ashley N.T., Weil Z.M., Nelson R.J. (2012). Inflammation: Mechanisms, costs, and natural variation. Ann. Rev. Ecol. Evol. Syst..

[68] Ferrero-Miliani L., Nielsen O.H., Andersen P.S., Girardin S.E. (2007). Chronic inflammation: Importance of NOD2 and NALP3 in interleukin-1β generation. Clin. Exp. Immunol..

[69] Villar J., Blanco J., Añón J.M., Santos-Bouza A., Blanch L., Ambrós A., Gandía F., Carriedo D., Mosteiro F., Basaldúa S. (2011). The ALIEN study: Incidence and outcome of acute respiratory distress syndrome in the era of lung protective ventilation. Intensive Care Med..

[70] Ware L.B., Matthay M.A. (2000). The acute respiratory distress syndrome. N. Engl. J. Med..

[71] Rubenfeld G.D., Caldwell E., Peabody E., Weaver J., Martin D.P., Neff M., Stern E.J., Hudson L.D. (2005). Incidence and outcomes of acute lung injury. N. Engl. J. Med..

[72] Slutsky A.S. (2002). Hot new therapy for sepsis and the acute respiratory distress syndrome. J. Clin. Investig..

[73] González-Reimers E., Santolaria-Fernández F., Martín-González M.C., Fernández-Rodríguez C.M., Quintero-Platt G. (2014). Alcoholism: A systemic proinflammatory condition. World J. Gastroenterol..

[74] Hirsh M.I., Junger W.G. (2008). Roles of heat shock proteins and γδT cells in inflammation. Am. J. Respir. Cell Mol. Biol..

[75] Weiss Y.G., Bromberg Z., Raj N., Raphael J., Goloubinoff P., Ben-Neriah Y., Deutschman C.S. (2007). Enhanced heat shock protein 70 expression alters proteasomal degradation of IκB kinase in experimental acute respiratory distress syndrome. Crit. Care Med..

[76] Chu E.K., Ribeiro S.P., Slutsky A.S. (1997). Heat stress increases survival rates in lipopolysaccharide-stimulated rats. Crit. Care Med..

[77] Bromberg Z., Raj N., Goloubinoff P., Deutschman C.S., Weiss Y.G. (2008). Enhanced expression of 70-kilodalton heat shock protein limits cell division in a sepsis-induced model of acute respiratory distress syndrome. Crit. Care Med..

[78] Smolen J.S., Breedveld F.C., Burmester G.R., Bykerk V., Dougados M., Emery P., Kvien T.K., Navarro-Compán M.V., Oliver S., Schoels M. (2016). Treating rheumatoid arthritis to target: 2014 update of the recommendations of an international task force. Ann. Rheum. Dis..

[79] Aletaha D., Neogi T., Silman A.J., Funovits J., Felson D.T., Bingham C.O., Birnbaum N.S., Burmester G.R., Bykerk V.P., Cohen M.D. (2010). 2010 rheumatoid arthritis classification criteria: An American College of Rheumatology/European League Against Rheumatism collaborative initiative. Arthritis Rheum..

[80] Klareskog L., Malmström V., Lundberg K., Padyukov L., Alfredsson L. (2011). Smoking, citrullination and genetic variability in the immunopathogenesis of rheumatoid arthritis. Semin. Immunol..

[81] Millar K., Lloyd S.M., McLean J.S., Batty G.D., Burns H., Cavanagh J., Deans K.A., Ford I., McConnachie A., McGinty A. (2013). Personality, socio-economic status and inflammation: Cross-sectional, population-based study. PLoS ONE.

[82] Callahan L.F., Pincus T. (1997). Education, self-care, and outcomes of rheumatic diseases: Further challenges to the “biomedical model” paradigm. Arthritis Rheum..

[83] Schett G., Redlich K., Xu Q., Bizan P., Gröger M., Tohidast-Akrad M., Kiener H., Smolen J., Steiner G. (1998). Enhanced expression of heat shock protein 70 (HSP70) and heat shock factor 1 (HSF1) activation in rheumatoid arthritis synovial tissue. Differential regulation of HSP70 expression and hsf1 activation in synovial fibroblasts by proinflammatory cytokines, shear stress, and antiinflammatory drugs. J. Clin. Investig..

[84] Kang E.H., Kim D.J., Lee E.Y., Lee Y.J., Lee E.B., Song Y.W. (2009). Downregulation of heat shock protein 70 protects rheumatoid arthritis fibroblast-like synoviocytes from nitric oxide-induced apoptosis. Arthritis Res. Ther..

[85] Van Roon J.A., van Eden W., van Roy J.L., Lafeber F.J., Bijlsma J.W. (1997). Stimulation of suppressive T cell responses by human but not bacterial 60-kD heat-shock protein in synovial fluid of patients with rheumatoid arthritis. J. Clin. Investig..

[86] Pockley A.G. (2003). Heat shock proteins as regulators of the immune response. Lancet.

[87] Anderton S.M., Van Der Zee R., Prakken B., Noordzij A., Van Eden W. (1995). Activation of T cells recognizing self 60-kD heat shock protein can protect against experimental arthritis. J. Exp. Med..

[88] Kaul G., Thippeswamy H. (2011). Role of heat shock proteins in diseases and their therapeutic potential. Indian J. Microbiol..

[89] Salinthone S., Ba M., Hanson L., Martin J.L., Halayko A.J., Gerthoffer W.T. (2007). Overexpression of human Hsp27 inhibits serum-induced proliferation in airway smooth muscle myocytes and confers resistance to hydrogen peroxide cytotoxicity. Am. J. Physiol. Lung Cell. Mol. Physiol..

[90] Lazaar A.L., Panettieri R.A. (2006). Airway smooth muscle as a regulator of immune responses and bronchomotor tone. Clin. Chest Med..

[91] Chiappara G., Gagliardo R., Siena A., Bonsignore M.R., Bousquet J., Bonsignore G., Vignola A.M. (2001). Airway remodelling in the pathogenesis of asthma. Curr. Opin. Allergy Clin. Immunol..

[92] Feigin V. (2016). Global, regional, and national life expectancy, all-cause mortality, and cause-specific mortality for 249 causes of death, 1980–2015: A systematic analysis for the Global Burden of Disease Study 2015. Lancet.

[93] Vos T., Allen C., Arora M., Barber R.M., Bhutta Z.A., Brown A., Carter A., Casey D.C., Charlson F.J., Chen A.Z. (2016). Global, regional, and national incidence, prevalence, and years lived with disability for 310 diseases and injuries, 1990–2015: A systematic analysis for the Global Burden of Disease Study 2015. Lancet.

[94] Wolff P.T., Arison L., Rahajamiakatra A., Raserijaona F., Niggemann B. (2012). High asthma prevalence and associated factors in urban malagasy schoolchildren. J. Asthma.

[95] Adeloye D., Chan K.Y., Rudan I., Campbell H. (2013). An estimate of asthma prevalence in Africa: A systematic analysis. Croatian Med. J..

[96] Bertorelli G., Bocchino V., Zhuo X., Chetta A., Del Donno M., Foresi A., Testi R., Olivieri D. (1998). Heat shock protein 70 upregulation is related to HLA-DR expression in bronchial asthma. Effects of inhaled glucocorticoids. Clin. Exp. Allergy.

[97] Changchun H., Haijin Z., Wenjun L., Zhenyu L., Dan Z., Laiyu L., Wancheng T., Shao-Xi C., Fei Z. (2011). Increased heat shock protein 70 levels in induced sputum and plasma correlate with severity of asthma patients. Cell Stress Chaperones.

[98] Vignola A.M., Chanez P., Polla B.S., Vic P., Godard P., Bousquet J. (1995). Increased expression of heat shock protein 70 on airway cells in asthma and chronic bronchitis. Am. J. Respir. Cell. Mol. Biol..

[99] Polla B.S., Bachelet M., Dall’Ava J., Vignola A.M. (1998). Heat shock proteins in inflammation and asthma: Dr Jekyll or Mr Hyde?. Clin. Exp. Allergy.

[100] Aron Y., Busson M., Polla B.S., Dusser D., Lockhart A., Swierczewski E., Favatier F. (1999). Analysis of HSP70 gene polymorphism in allergic asthma. Allergy.

[101] Wong H.R., Wispe J.R. (1997). The stress response and the lung. Am. J. Physiol. Lung Cell. Mol. Physiol..

[102] Shingai R., Maeda T., Onishi S., Yamamoto Y. (1995). Autonatibody against 70 kD heat shock protein in patients with autoimmune liver diseases. J. Hepatol..

[103] Xu Q., Kiechl S., Mayr M., Metzler B., Egger G., Oberhollenzer F., Willeit J., Wick G. (1999). Association of serum antibodies to heat-shock protein 65 with carotid atherosclerosis. Circulation.

[104] Salman A.N. (2015). Serum Levels Evaluation of Heat Shock Protein70 during Gestation and Fetal Birth Weight in Asthmatic Women of Thi-QAR Province, Iraq. J. Al-Nahrain Univ..

[105] Schatz M. (2009). Is maternal asthma a life or death issue for the baby?. Thorax.

[106] Yang M., Wu T., Cheng L., Wang F., Wei Q., Tanguay R.M. (2005). Plasma antibodies against heat shock protein 70 correlate with the incidence and severity of asthma in a Chinese population. Respir. Res..

[107] PeRIšIć T., Srećković M., Matić G. (2007). Changes of antioxidant enzyme activity and heat shock protein content in lymphocytes of children with asthma. Arch. Biol. Sci..

[108] Tong W., Luo W. (2000). Heat shock proteins’ mRNA expression in asthma. Respirology.

[109] Karin M., Greten F.R. (2005). NF-[kappa] B: Linking inflammation and immunity to cancer development and progression. Nat. Rev. Immunol..

[110] Torre L.A., Bray F., Siegel R.L., Ferlay J., Lortet-Tieulent J., Jermal A. (2015). Global cancer statistics 2012. CA Cancer J. Clin..

[111] Hanahan D., Weinberg R.A. (2000). The hallmarks of cancer. Cell.

[112] Calderwood S.K., Stevenson M.A., Murshid A. (2012). Heat shock proteins, autoimmunity, and cancer treatment. Autoimmun. Dis..

[113] Kapoor C., Vaidya S. (2013). Heat shock protein (HSP) and cancer: An overview. Am. J. Med. Dent. Sci..

[114] Beere H.M., Green D.R. (2001). Stress management–heat shock protein-70 and the regulation of apoptosis. Trends Cell Biol..

[115] Wu J., Liu T., Rios Z., Mei Q., Lin X., Cao S. (2017). Heat shock proteins and cancer. Trends Pharmacol. Sci..

[116] Vergara D., Simeone P., del Boccio P., Toto C., Pieragostino D., Tinelli A., Acierno R., Alberti S., Salzet M., Giannelli G. (2013). Comparative proteome profiling of breast tumor cell lines by gel electrophoresis and mass spectrometry reveals an epithelial mesenchymal transition associated protein signature. Mol. BioSyst..

[117] Shiota M., Bishop J.L., Nip K.M., Zardan A., Takeuchi A., Cordonnier T., Beraldi E., Bazov J., Fazli L., Chi K. (2013). Hsp27 regulates epithelial mesenchymal transition, metastasis, and circulating tumor cells in prostate cancer. Cancer Res..

[118] Lianos G.D., Alexiou G.A., Mangano A., Mangano A., Rausei S., Boni L., Dionigi G., Roukos D.H. (2015). The role of heat shock proteins in cancer. Cancer Lett..

[119] Jego G., Hazoumé A., Seigneuric R., Garrido C. (2013). Targeting heat shock proteins in cancer. Cancer Lett..

[120] Chaiwatanasirikul K.A., Sala A. (2011). The tumour-suppressive function of CLU is explained by its localisation and interaction with HSP60. Cell Death Dis..

[121] Ghosh J.C., Siegelin M.D., Dohi T., Altieri D.C. (2010). Heat shock protein 60 regulation of the mitochondrial permeability transition pore in tumor cells. Cancer Res..

[122] Chalmin F., Ladoire S., Mignot G., Vincent J., Bruchard M., Remy-Martin J.P., Boireau W., Rouleau A., Simon B., Lanneau D. (2010). Membrane-associated Hsp72 from tumor-derived exosomes mediates STAT3-dependent immunosuppressive function of mouse and human myeloid-derived suppressor cells. J. Clin. Investig..

[123] Trinh D.L., Elwi A.N., Kim S.W. (2010). Direct interaction between p53 and Tid1 proteins affects p53 mitochondrial localization and apoptosis. Oncotarget.

[124] Spooner R., Yilmaz Ö. (2011). The role of reactive-oxygen-species in microbial persistence and inflammation. Int. J. Mol. Sci..

[125] Simon H.U., Haj-Yehia A., Levi-Schaffer F. (2000). Role of reactive oxygen species (ROS) in apoptosis induction. Apoptosis.

[126] Fischer R., Maier O. (2015). Interrelation of oxidative stress and inflammation in neurodegenerative disease: Role of TNF. Oxid. Med. Cell. Longev..

[127] Nowsheen S., Yang E.S. (2012). The intersection between DNA damage response and cell death pathways. Exp. Oncol..

[128] Nita M., Grzybowski A. (2016). The role of the reactive oxygen species and oxidative stress in the pathomechanism of the age-related ocular diseases and other pathologies of the anterior and posterior eye segments in adults. Oxid. Med. Cell. Longev..

[129] Barnes P.J. (1990). Reactive oxygen species and airway inflammation. Free Radic. Biol. Med..

[130] Henricks P.A., Nijkamp F.P. (2001). Reactive oxygen species as mediators in asthma. Pulm. Pharmacol. Ther..

[131] Winrow V.R., McLean L., Morris C.J., Blake D.R. (1990). The heat shock protein response and its role in inflammatory disease. Ann. Rheum. Dis..

[132] Chen Y., Voegeli T.S., Liu P.P., Noble E.G., Currie R.W. (2007). Heat shock paradox and a new role of heat shock proteins and their receptors as anti-inflammation targets. Inflamm. Allergy Drug Targets.

[133] Hauet-Broere F., Wieten L., Guichelaar T., Berlo S., Van der Zee R., Van Eden W. (2006). Heat shock proteins induce T cell regulation of chronic inflammation. Ann. Rheum. Dis..

[134] Tóth M.E., Gombos I., Sántha M. (2015). Heat shock proteins and their role in human diseases. Acta Biol. Szeged..

[135] Arnal M.E., Lallès J.P. (2016). Gut epithelial inducible heat-shock proteins and their modulation by diet and the microbiota. Nutr. Rev..

[136] Goldstein M.G., Li Z. (2009). Heat-shock proteins in infection-mediated inflammation-induced tumorigenesis. J. Hematol. Oncol..

[137] Hatfield P.D., Lovas S. (2012). Role of Hsp70 in cancer growth and survival. Protein Pept. Lett..

[138] Chauhan D., Li G., Hideshima T., Podar K., Mitsiades C., Mitsiades N., Catley L., Tai Y.T., Hayashi T., Shringarpure R. (2003). Hsp27 inhibits release of mitochondrial protein Smac in multiple myeloma cells and confers dexamethasone resistance. Blood.

[139] Nylandsted J., Rohde M., Brand K., Bastholm L., Elling F., Jäättelä M. (2000). Selective depletion of heat shock protein 70 (HSP70) activates a tumor-specific death program that is independent of caspases and bypasses Bcl-2. Proc. Natl. Acad. Sci. USA.

[140] Zininga T., Shonhai A. (2014). Are heat shock proteins druggable candidates?. Am. J. Biochem. Biotech..

[141] Henstridge D.C., Febbraio M.A., Hargreaves M. (2016). Heat shock proteins and exercise adaptations. Our knowledge thus far and the road still ahead. J. Appl. Physiol..

[142] Noble E.G., Shen G.X. (2012). Impact of exercise and metabolic disorders on heat shock proteins and vascular inflammation. Autoimmun. Dis..

[143] Naughton L., Lovell R., Madden L. (2006). Heat shock proteins in exercise: A review. J. Exerc. Sci. Physiother..

